# Neuroendocrine tumours of the ampulla of Vater: clinico-pathological features, surgical approach and assessment of prognosis

**DOI:** 10.1007/s00423-012-0951-7

**Published:** 2012-04-03

**Authors:** Traian Dumitrascu, Simona Dima, Vlad Herlea, Victor Tomulescu, Mihnea Ionescu, Irinel Popescu

**Affiliations:** 1 Department of Surgery, Center of General Surgery and Liver Transplantation, Fundeni Clinical Institute, Fundeni Street no 258, 022328 Bucharest, Romania; 2 Department of Pathology, Center of General Surgery and Liver Transplantation, Fundeni Clinical Institute, Fundeni Street no 258, 022328 Bucharest, Romania

**Keywords:** Ampulla of Vater, Neuroendocrine tumour, Lymph nodes metastases, Pancreaticoduodenectomy, Outcome

## Abstract

**Background/aims:**

Neuroendocrine tumours occur very rarely in the ampulla of Vater and their clinical behaviour is unknown. The aim of this study is to assess the clinico-pathological features, surgical approach and prognosis of these patients.

**Methods:**

Six patients with neuroendocrine tumours of the ampulla of Vater treated with curative intent surgery at a single centre were retrospectively analysed. A univariate analysis of potential prognostic factors was also performed (data provided from the present study and literature review).

**Results:**

Pancreaticoduodenectomy was curative in all the patients. Overall and disease-free survival rates were significantly better for G1/G2 tumours (*p* = 0.006 and *p* = 0.004, respectively). Although frequent, lymph node metastases did not influenced both overall (*p* = 0.760) and disease-free survival rates (*p* = 0.745). No significant differences of survival were observed in patients with ENETS stage I/II disease, as compared to ENETS stage III disease (*p* = 0.169 and *p* = 0.137, respectively). No differences were observed according to UICC staging system (*p* = 0.073 and *p* = 0.177, respectively). Tumours that are less than 2 cm or limited to the ampulla appear to have a better prognosis.

**Conclusion:**

The WHO 2010 classification appear to accurately predict patient prognosis, while the ENETS or UICC staging systems have a limited value (especially in regard to lymph node metastases). Radical surgery (i.e. pancreaticoduodenectomy with lymphadenectomy) should be the standard approach in most patients with NET of the ampulla of Vater because this procedure removes all the potential tumour-bearing tissue.

## Introduction

Neuroendocrine tumours (NET) of the ampulla of Vater are considered to be an exceptional pathology. A total of 139 NET of the ampulla of Vater (carcinoids and high-grade neuroendocrine carcinomas) were identified between 1973 and 2006 in a recent analysis of the Surveillance, Epidemiology and End Results Program of the National Cancer Institute [1]. To date, data in the literature regarding NET of the ampulla of Vater consist only of case reports [2, 3] or very small case series [4–8].

Currently, there is no standard of care for NET of the ampulla of Vater [3], mostly due to their poorly defined natural history [4]. Therapy for these tumours varies from local resection (endoscopic or surgical ampullectomy) [9–12] to aggressive surgery (i.e. pancreaticoduodenectomy with lymph node dissection) [4, 5, 13]. The prognostic value of locoregional lymph node metastasis or other factors, such as depth of invasion or tumour diameter, are not clearly established [1, 4]. Thus, the question of the best therapeutic approach remains open.

This study reports a single-centre experience of six patients diagnosed with NET of the ampulla of Vater, staged according to the 2010 World Health Organisation (WHO) classification [14] and tumour-nodes-metastasis (TNM) staging [15, 16] (Table 1). The clinical and pathological findings and the surgical treatment of the patients included in the study are discussed. The relevant literature was reviewed. Furthermore, an analysis of potential factors influencing survival after curative intent surgery for NET of the ampulla of Vater was performed based on data from the present study and available data from the literature.

**Table 1:** Staging systems for NET of the ampulla of Vater: WHO [14], ENETS [16] and UICC [15]

	WHO classification							
	NN grade 1 (low grade)				<2 mitoses/10 HPF and < 3 % Ki67			
	NN grade 2 (intermediate grade)				2–20 mitoses/10 HPF or 3 %–20 % Ki67			
	NC grade 3 (high grade)				> 20 mitoses/10 HPF or > 20 % Ki67			
	ENETS staging system				UICC staging system			
T—primary tumour								
Tx	Primary tumour cannot be assessed							
T0	No evidence of primary tumour							
T1	Invasion of lamina propria or submucosa and size ≤ 1 cm				Limited to ampulla of Vater or sphincter of Oddi			
T2	Invasion of muscularis propria or size > 1 cm				Invasion of the duodenum wall			
T3	Invasion of the pancreas or retroperitoneum				Invasion of the pancreas			
T4	Invasion of the peritoneum or other organs				Invasion in peripancreatic soft tissues or other adjacent organs or structures			
N—regional lymph nodes								
Nx	Regional lymph nodes cannot be assessed							
N0	No regional lymph node metastasis							
N1	Regional lymph node metastasis present							
M—distant metastasis								
Mx	Distant metastasis cannot be assessed							
M0	No distant metastasis							
M1	Distant metastasis present							
Staging	Stage I	T1	N0	M0	Stage Ia	T1	N0	M0
	Stage IIa	T2	N0	M0	Stage Ib	T2	N0	M0
	Stage IIb	T3	N0	M0	Stage IIa	T3	N0	M0
	Stage IIIa	T4	N0	M0	Stage IIb	T1,2,3	N1	M0
	Stage IIIb	Any T	N1	M0	Stage III	T4	Any N	M0
	Stage IV	Any T	Any N	M1	Stage IV	Any T	Any N	M1

*WHO* World Health Organisation classification, *ENETS* European Neuroendocrine Tumour Society staging system, *UICC* International Union Against Cancer staging system, *NN* neuroendocrine neoplasm, *NC* neuroendocrine carcinoma, *HPF* high power fields

## Patients and methods

All patients with a final pathological diagnosis of NET of the ampulla of Vater were retrospectively identified from a prospectively gathered electronic database established at our Department. Between 2002 and 2011 (August 1), 128 patients with malignant tumours of the ampulla of Vater underwent pancreaticoduodenectomy at the Center of General Surgery and Liver Transplantation, Fundeni Clinical Institute, Bucharest, Romania. Out of these, 120 tumours were diagnosed as adenocarcinoma (93.8 %), 2 tumours were diagnosed as gastrointestinal stromal tumours (1.62 %) and the remaining 6 patients were diagnosed with NET (4.6 %).

Patient data included age at diagnosis, sex, presenting symptoms, associated pathology, preoperative imaging and biopsy results, type of operation, pathology and follow-up with survival status.

Computed tomography (CT) and/or a magnetic resonance imaging (MRI) were the main preoperative imaging work-up modalities, with the aim of characterizing the tumour and assessing the presence of involved locoregional lymph nodes or distant metastases.

The pathological data were assessed by the same pathologist (VH), considering the WHO 2010 classification, the European Neuroendocrine Tumour Society (ENETS) and International Union Against Cancer (UICC) staging systems. All the tumours were immunostained for chromogranin A, synaptophysin and neuron-specific enolase to confirm the diagnosis of NET. For grading, the Ki67 index was assessed using the MIB1 antibody as a percent of 2,000 tumour cells in areas where the highest nuclear labelling was noticed.

Follow-up protocol after surgery included clinical examination and CT or MRI every 3 months in the first year and every 6 months after the first year.

## Survival analysis

Because the number of the patients in the present series was too small to perform a survival analysis and to assess the potential factors influencing the long-term outcome after curative intent surgery for NET of the ampulla of Vater, we searched the literature for additional available data (i.e. WHO classification, ENETS and UICC staging systems, tumour diameter, T stage, lymph node involvement, survival status, overall and disease-free survival). A total of 37 patients who underwent pancreaticoduodenectomy for NET of the ampulla of Vater were introduced in a univariate analysis, including our cases (Table 2) [4, 5, 8, 17–21].

**Table 2:** Data of 37 patients with pancreaticoduodenectomy for NET of the ampulla of Vater, taken into consideration for survival analysis

No	Reference	WHO	ENETS	UICC	*D*	T (ENETS)	N	DFS	OS	Status
1	Nassar H et al. [8]	NC G3	IIIb	IIb	–	–	N1	24	30	Dead
2	Nassar H et al. [8]	NC G3	IIIb	IIb	–	–	N1	10	13	Dead
3	Nassar H et al. [8]	NC G3	IIIb	IIb	–	–	N1	17	17	Alive
4	Nassar H et al. [8]	NC G3	IIIb	III	–	–	N1	10	16	Dead
5	Nassar H et al. [8]	NC G3	IIIb	III	–	–	N1	10	10	Alive
6	Nassar H et al. [8]	NC G3	IIIb	III	–	–	N1	2	4	Dead
7	Nassar H et al. [8]	NC G3	IIb	IIa	–	–	N0	48	48	Alive
8	Nassar H et al. [8]	NC G3	IIb	Ib	–	–	N0	6	6	Alive
9	Carter JT et al. [4]	NC G3	IIIb	IIb	3.5	T3	N1	10	15	Dead
10	Carter JT et al. [4]	NT G1/G2	IIIb	IIb	1	T1	N1	48	48	Alive
11	Carter JT et al. [4]	NT G1/G2	IIIb	IIb	1.6	T2	N1	31	31	Alive
12	Carter JT et al. [4]	NT G1/G2	IIa	Ia	2.5	T2	N0	25	25	Alive
13	Carter JT et al. [4]	NT G1/G2	IIIb	IIb	2.1	T2	N1	19	19	Alive
14	Hwang S et al. [5]	–	IIIa	Ib	2	T4	N0	6	9	Dead
15	Hwang S et al. [5]	–	IIIa	IIa	2.3	T4	N0	6	16	Dead
16	Hwang S et al. [5]	–	I	Ia	1	T1	N0	37	42	Dead
17	Hwang S et al. [5]	–	IIIa	IIa	3.5	T4	N0	31	31	Dead
18	Hwang S et al. [5]	–	IIIb	IIb	5	T4	N1	30	44	Alive
19	Hwang S et al. [5]	–	IIa	Ia	1.6	T2	N0	38	38	Alive
20	Hwang S et al. [5]	–	I	Ia	1	T1	N0	35	35	Alive
21	Hwang S et al. [5]	–	I	Ia	0.7	T1	N0	29	29	Alive
22	Hwang S et al. [5]	–	IIIb	IIb	1.5	T2	N1	21	21	Alive
23	Hwang S et al. [5]	–	IIIa	Ib	2.7	T4	N0	13	20	Alive
24	Selvakumar E et al. [19]	NT G1	–	–	–	–	–	24	24	Alive
25	Selvakumar E et al. [19]	NC G3	–	–	–	–	–	9	13	Dead
26	Selvakumar E et al. [19]	NC G3	–	–	–	–	–	7	11	Dead
27	Selvakumar E et al. [19]	NC G3	–	–	–	–	–	4	7	Dead
28	Cavazza A et al. [17]	NC G3	IIIb	IIb	3	T3	N1	2	8	Dead
29	Stojsic Z et al. [21]	NC G3	IIIb	IIb	3	T2	N1	2	11	Alive
30	Huang S et al. [18]	NC G3	IIIb	IIb	2.8	T3	N1	5	10	Dead
31	Senda E et al. [20]	NT G2	IIIb	IIb	0.7	T2	N1	24	24	Alive
32	Present study	NT G2	IIIb	IIb	0.8	T1	N1	80	80	Alive
33	Present study	NC G3	I	Ia	1	T1	N0	18	24	Dead
34	Present study	NC G3	IIIb	IIb	1.5	T2	N1	52	52	Alive
35	Present study	NT G1	IIa	Ia	2	T2	N0	32	32	Alive
36	Present study	NT G1	IIIb	IIb	2	T3	N1	6	6	Alive
37	Present study	NT G1	IIIb	IIb	1	T1	N1	4	4	Alive

*WHO* World Health Organisation classification, *ENETS* European Neuroendocrine Tumour Society staging system, *UICC* International Union Against Cancer staging system, *D* tumour maximal diameter (cm), *T* tumour status, *N* locoregional lymph nodes status, *DFS* disease-free survival (months), *OS* overall survival (months), *NT* neuroendocrine tumour, *NC* neuroendocrine carcinoma

### Statistical analysis

Numeric data are expressed as median (range). Survival curves were estimated using the Kaplan–Meier method and were compared using the log-rank test. A *p* value < 0.05 was considered to be statistically significant.

## Results

### Patient demographics and preoperative work-up

The patients were predominantly male (male/female ratio = 4:2), with a median age of 54 years (range, 32–68 years). The main presenting symptom was jaundice (four patients), while two patients presented with epigastric pain. Five patients presented with cholestasis syndrome (minor cholestasis in one patient, major cholestasis in four patients). In the patients with jaundice, the total bilirubin level ranged from 3.0 to 17.0 mg/dl (median 13 mg/dl). No patient had anaemia.

**Fig. 1:**
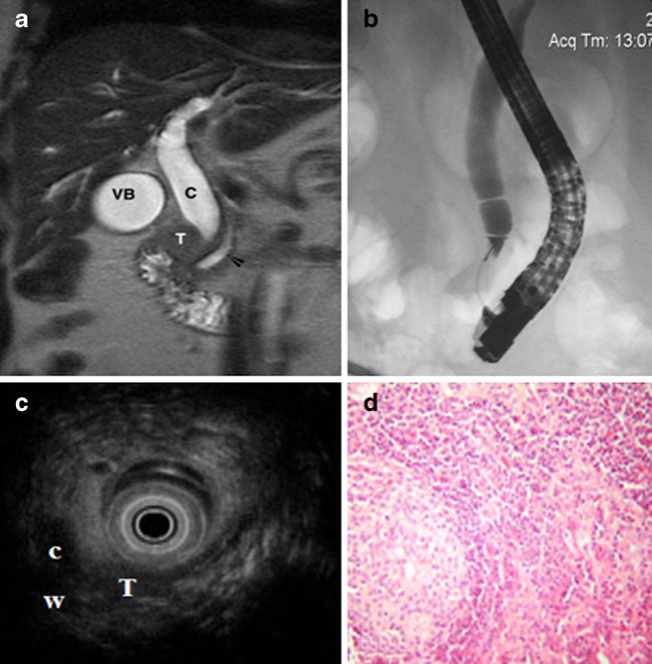
**a** Magnetic resonance cholangiopancreatography showing an ampullary soft tissue mass (*T*), with secondary dilatation of the common bile duct (*c*) and main pancreatic duct (*arrow head*); **b** endoscopic retrograde cholangiopancreatography showing an irregular, fusiform stenosis of the common bile duct toward the ampulla of Vater, with secondary dilatation; **c** endoscopic ultrasound revealing the ampulla with a 8 × 7 mm hypoechoic lesion (*T*), not invading the muscularis propria and with secondary dilatation of the common bile duct (*c*) and Wirsung's duct (*W*); **d** histological features (hematoxylin-eosin, original magnification ×40) revealing metastases into a locoregional lymph node of a G1 neuroendocrine neoplasm—nests of uniforms, polygonal tumour cells with round nuclei and “salt and pepper” chromatin

Preoperative imaging included CT (two patients), MRI (three patients) or both (one patient). The CT and/or MRI showed the ampullary tumour in all cases; in patients with cholestasis syndrome, on noticed dilatation of the intra- and extrahepatic bile ducts, along with dilatation of Wirsung's duct (Fig. 1a). No distant metastases to the liver, lung, peritoneum or other organs were observed. Enlarged locoregional lymph nodes were observed in one patient on preoperative imaging.

Endoscopic ultrasound was performed on one patient, showing a hypoechoic tumour mass at the level of the ampulla of Vater (0.8 cm, not invading the muscularis propria), with secondary dilatation of the common bile duct and Wirsung's duct. No enlarged lymph nodes were detected (Fig. 1c).

Upper endoscopy with biopsy was performed in four patients. Preoperative tumour biopsy diagnosed a NET in three patients; in one patient, although malignancy was suspected, it was not possible to differentiate the tumour histopathologically. Endoscopic retrograde cholangiography was performed in one patient, showing a dilated common bile duct with distal stenosis (Fig. 1b). None of the patients had symptoms of an endocrine hypersecretion syndrome, but one patient had associated neurofibromatosis (von Recklinghausen's disease).

### Operative treatment and pathological findings

All the patients were treated with pylorus-preserving pancreaticoduodenectomy, with nil mortality. All the operative specimens were assessed as R0 resections (without microscopic residual tumour). Median size of the tumour was 1.25 cm (range, 0.8 to 2 cm). All of the tumours were found to be diffusely positive for chromogranin A, synaptophysin and neuron-specific enolase with immunohistochemistry, confirming their neuroendocrine origin. The Ki67 index was ≤ 2 % in three tumours, 3–20 % in one tumour and > 20 % in two tumours.

The median number of harvested lymph nodes was 15 (range, 10–19). Locoregional lymph node metastases were observed in four patients. Lymph node ratio (i.e. ratio of positive to excised lymph nodes) ranged from 0.06 to 0.68. The presence of lymph node metastases was assessed by histopathological examination using hematoxylin-eosin staining (Fig. 1d).

Tumours assessments according to the ENETS, UICC staging systems and the WHO 2010 classification are shown in Table 2.

### Follow-up and survival status

Complete follow-up data were available for all patients, as shown in Table 2. No patient had adjuvant chemotherapy. At the last follow-up (November 1, 2011), five patients were alive and disease-free, while one patient died at 24 months after surgery with liver metastases. Interestingly, the deceased patient's tumour was assessed as a stage I tumour according to ENETS/UICC TNM classification (1-cm tumour size, confined to the submucosa and no lymph node metastases), although it was a high-grade neuroendocrine carcinoma.

### Factors influencing long-term survival after curative intent surgery (i.e. pancreaticoduodenectomy) for NET of the ampulla of Vater (present study and data from the literature)

Patients included in the survival analysis had the following features (Table 2): G3 neuroendocrine carcinoma was found in 17 patients (63 %) and G1/G2 neuroendocrine neoplasms were found in 10 patients. Twenty patients had lymph node metastases (60 %) and 13 patients had negative lymph nodes. Tumours were limited to the ampulla (T1/T2 ENETS) in 15 patients (60 %) and were locally invasive (T3/T4 ENETS) in 10 patients. Tumour diameter was <2 cm in 12 patients (48 %) and was ≥2 cm in 13 patients. ENETS stage III disease was found in 24 patients (73 %) and ENETS stages I and II disease was found in 9 patients. UICC stage III disease was found in 3 patients, while UICC stage I and II diseases were found in 8 patients and 22 patients (66 %), respectively.

**Fig. 2:**
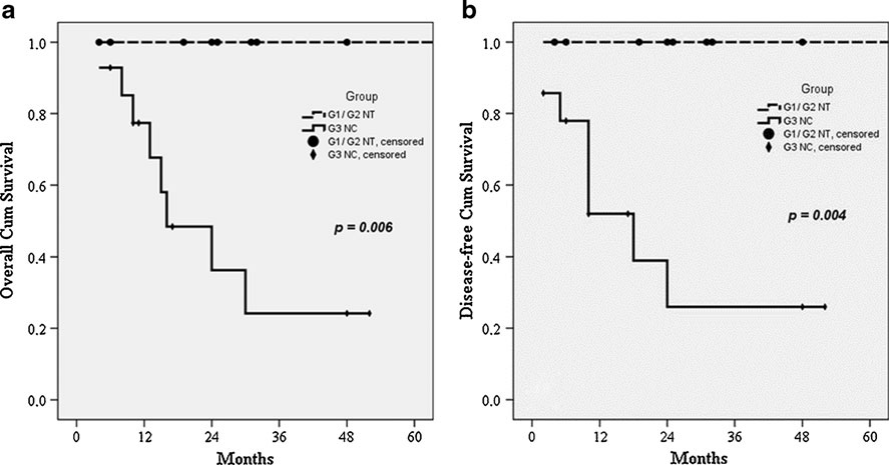
Overall (**a**) and disease-free (**b**) survival curves according to WHO classification for 27 patients undergoing pancreaticoduodenectomy for NET of the ampulla of Vater

**Fig. 3:**
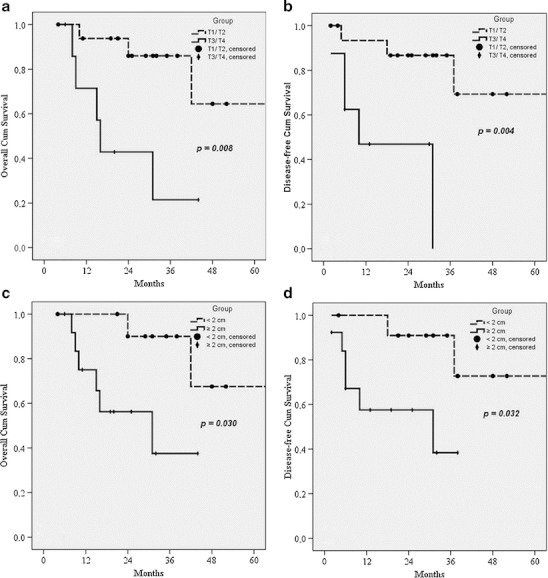
Overall (**a**) and disease-free (**b**) survival curves according to local invasiveness of the tumour for 25 patients undergoing pancreaticoduodenectomy for NET of the ampulla of Vater (tumours limited to ampulla—T1/T2 vs. local invasive tumours—T3/T4); overall (**c**) and disease-free (**d**) survival curves according to tumour diameter for 25 patients undergoing pancreaticoduodenectomy for NET of the ampulla of Vater (<2 cm vs. ≥2 cm)

**Figure 4:**
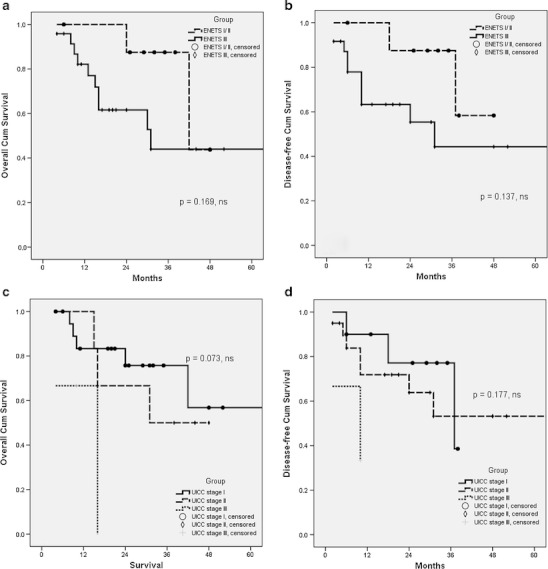
Overall (**a**) and disease-free (**b**) survival curves according to ENETS staging system for 33 patients undergoing pancreaticoduodenectomy for NET of the ampulla of Vater (stage I/II vs. stage III); overall (**c**) and disease-free (**d**) survival curves according to UICC staging system for 33 patients undergoing pancreaticoduodenectomy for NET of the ampulla of Vater (stage I vs. II vs. stage III)

WHO classification was found to be a significant factor for predicting both overall and disease-free survival (Table 3), as shown in Fig. 2a, b. Interestingly, there were no significant differences in terms of overall and disease-free survival in patients with lymph node metastases, as compared with patients who had negative lymph nodes (Table 3). Depth of the tumour invasion (T status) appeared to be a significant factor influencing overall and disease-free survival (Table 3), as shown in Fig. 3a, b. Regarding tumour diameter, the overall and disease-free median survival for patients with tumours <2 cm was significantly higher than in patients with tumours ≥2 cm (Table 3), as shown in Fig. 3c, d. There were no significant differences in overall and disease-free survival among patients with ENETS stage I/II vs. stage III disease (Table 3), as shown in Fig. 4a, b. No differences of both overall and disease-free survival rates were observed between patients with UICC stages I, II and III (Table 3), as shown in Fig. 4c, d.

**Table 3:** Univariate predictors of overall and disease-free survival in patients with pancreaticoduodenectomy for NET of the ampulla of Vater

	Median OS (months)	*P* value	Median DFS (months)	*P* value
WHO classification				
G1/G2	NA^a^	0.006	NA^a^	0.004
G3	NA^a^		NA^a^	
Lymph nodes				
N0	42	0.760, ns	37	0.0745, ns
N1	50		50	
ENETS				
T1/T2	63	0.008	63	0.004
T3/T4	22		17	
Tumour diameter				
<2 cm	65	0.030	66	0.032
≥2 cm	27		23	
ENETS stage				
Stage I/II	42	0.169, ns	41	0.137, ns
Stage III	45		43	
UICC stage				
Stage I	42	0.073, ns	37	0.177, ns
Stage II	51		49	
Stage III	16		10	

*OS* overall survival, *DFS* disease-free survival, *WHO* World Health Organisation classification, *ENETS* European Neuroendocrine Tumour Society staging system, *UICC* International Union Against Cancer staging system

^a^Data for median survival were not available because all of the patients in the G1/G2 group were censored

## Discussions

Although NET (initially assessed as carcinoid tumour) was first described over 100 years ago at the University of Munich by Siegfried Oberndorfer, these tumours still raise many issues regarding classification, prognosis and choice of the best therapeutic approach [22].

The incidence and prevalence of NET seems to have increased in recent years, most likely due to an improvement in diagnostic techniques [23, 24]. However, among gastroenteropancreatic NET, the ampulla of Vater represents an uncommon site for the disease [23]. Nevertheless, the number of papers addressing the ampulla of Vater NET seems to have increased in recent years [1, 4–6, 19]. Thus, the choice of an optimal approach for this pathology remains difficult.

Gastroenteropancreatic NET are, by far, less frequent than adenocarcinomas [24], a statement that is also true for NET of the ampulla of Vater [19, 25]. In a recent analysis of 450 patients with ampullary neoplasms resected at Johns Hopkins, none of the tumours were diagnosed as neuroendocrine [26]. Other series reported an incidence of NET that ranged from less than 1 to 8.8 % (of all patients undergoing resections of malignant tumours of the ampulla of Vater) [1, 5, 19, 25].

CT, MRI, ultrasound endoscopy and endoscopic retrograde cholangiopancreatography with biopsy are the main tools for preoperatively assessing NET of the ampulla of Vater [3]. Accuracy rates of biopsy for the preoperative diagnosis of NET range from to 14 to 66 % [3–6]. In this series, a correct diagnosis of a NET was established preoperatively in three patients (out of the four patients who had a preoperative biopsy). Thus, the preoperative diagnostic accuracy was 75 %.

Currently, several options are available to treat NET of the ampulla of Vater. Endoscopic local resection and surgical ampullectomy have been considered to be safe for small NET of the ampulla of Vater (less than 2 cm) [27] or in patients with severe comorbidities [3]. Endoscopic ampullectomy is considered to be safe only in very selected cases of well-differentiated NET (T1 N0, negative resection margins, Ki67 <2 %) [12]. A major limitation of these procedures is the inability to address locoregional lymph node metastases, possibly leading to an inadequate oncological operation and potentially jeopardizing the patient's prognosis [28].

Pancreaticoduodenectomy has been generally considered the procedure of choice for NET of the ampulla of Vater that are larger than 2 cm and for cases of neuroendocrine carcinomas [3, 6]. In a review of 105 reported cases from the literature, pancreaticoduodenectomy was performed in more than 50 % of the patients [3]; a preference for this surgical approach was also noticed in the most recent reported series [4–6]. In a recent multi-institutional review of patients with resected small neuroendocrine pancreatic and periampullary NET, local resection had similar results to pancreaticoduodenectomy in terms of overall survival but was associated with significantly lower morbidity [29]. Similar data were reported by previous studies [30]. However, this study addressed a selected group of patients (with tumours less than 3 cm and no involved lymph nodes or liver metastases) [29]. Moreover, in experienced centres, the morbidity and mortality rates of the transduodenal local excision of ampullary lesions are comparable to pancreaticoduodenectomy [31].

In clinical practice, it is impossible to differentiate a neuroendocrine neoplasm from a neuroendocrine carcinoma intraoperatively. Sometimes, it is very difficult to differentiate NET from an adenocarcinoma of the ampulla of Vater on frozen sections [3]. Moreover, preoperative imaging tools or intraoperative exploration may not identify liver metastases that are smaller than 0.5 cm or lymph node metastases [24]. The lack of an accurate assessment of small liver or lymph node metastases may jeopardize the choice for an adequate surgical approach.

A question that arises is: does the therapeutic approach to the NET of the ampulla of Vater have the same principles of adenocarcinoma? Pancreaticoduodenectomy represents the procedure of choice for adenocarcinoma of the ampulla of Vater [26] and has proved to be curative in the majority of patients without lymph node metastases [32]. Ampullectomy is recommended only in highly selected cases—Tis/T1 N0, G1—G2 tumours [25, 28]. Moreover, even in T1 tumours, the rate of lymph node metastases is approximately 10 %; thus, in patients with a pTis or pT1 carcinoma, ampullectomy should be accomplished by a local lymph node dissection to ensure an oncological resection [25].

For ampullary adenocarcinomas, the presence of metastases in locoregional lymph nodes is a poor prognostic factor that is encountered in 47 to 65 % of the resected patients [25, 26, 28, 32]. An increased rate of metastases in the locoregional lymph nodes (up to 80 %) was also observed in patients with NET of the ampulla of Vater resected by pancreaticoduodenectomy, especially in patients with high-grade carcinomas [1, 8], but no correlation with survival was found [4, 6]. Interestingly, even in small tumours (less than 2 cm), the presence of lymph node metastases is higher than 50 % [4, 7, 30, 33]. However, a recent study from Asan Medical Center showed an incidence of positive lymph nodes of only 20 % after curative intent resection of NET of the ampulla of Vater [5].

In this series of patients, a high incidence of locoregional lymph node metastases was observed, although none of the tumours were larger than 2 cm (median size—1.25 cm). However, no correlation with survival was identified for the involved lymph nodes. The four patients with histologically proven positive lymph nodes survived without recurrence for up to 80 months, while a patient without lymph node metastases died 2 years after surgery with recurrent disease in the liver. Interestingly, preoperative imaging failed to detect metastases in the locoregional lymph nodes in most patients and in patients with pathologically confirmed lymph node metastases; intraoperative exploration did not show macroscopically enlarged lymph nodes. Conversely, the enlarged lymph nodes detected on preoperative CT in one patient were not pathologically confirmed as metastases. The same features were reported by other studies [4].

In summary, although an increased rate of positive lymph nodes was reported in both adenocarcinomas and NET of the ampulla of Vater, the clinical impact of this finding appears to be different in the two pathologies.

Assessment of prognosis is another important issue for NET of the ampulla of Vater. NET of the ampulla of Vater has a very similar prognosis to the prognosis of gastroenteropancreatic NET in general, with a 5-year survival rate of approximately 70 % [7]. Some studies reported a worse prognosis for NET of the ampulla of Vater compared to duodenal NET [33]. According to data from the Surveillance, Epidemiology and End Results Program, survival rates at 5 and 10 years are 82 and 71 % for NET, respectively, while for high-grade neuroendocrine carcinomas of the ampulla of Vater, the prognosis is significantly worse (15 % at 5 and 10 years) [1, 8]. The recurrence rate is approximately 40 to 50 % [3, 5], with the liver being the most common site of initial metastases [5].

Assessment of prognosis in patients with NET of the ampulla of Vater is difficult due to the small number of patients reported in the literature [3]. The most widely used classification was proposed by UICC [15]. In 2010, the WHO proposed a new pathological classification for gastroenteropancreatic NET [14] and the ENETS previously (2006) proposed a grading system along with a TNM staging [16]. The main goal of these classifications was to better assess the prognosis of patients with different tumour locations. Although ENETS provides a TNM staging system for NET of the ampulla of Vater [16], its clinical value is unknown because there are no large series of patients to provide survival data.

In the present study, tumour grading (WHO 2010) classification seems to accurately predict the prognosis of patients with NET of the ampulla of Vater, while the ENETS or UICC staging systems does not appear to be correlated with survival. Previous studies showed that the ENETS staging system is not a good predictor of prognosis for patients with NET of the ampulla of Vater [4]; however, high-grade neuroendocrine carcinoma has been associated with a poor prognosis and a median survival between 10.3 and 14.5 months [1, 6, 8, 19]. Thus, an advanced TNM stage is not necessarily correlated with a worse survival rate. The present study showed a correlation of the tumour size with the recurrence rate and overall survival, contrary to data from previous studies [34]. Tumour diameter (more than 2 cm) and tumour extension beyond the ampulla were also found to be associated with a high risk for recurrence upon univariate analysis in a recent study [5]. Although certain factors, such as younger age [1, 6], small tumour size [1, 6, 8] and the presence of type I neurofibromatosis [1] seem to be more frequent in patients with G1/G2 NET of the ampulla of Vater, these data cannot be used in clinical decision making. Angiolymphatic, perineural or venous invasion was recently found to be more frequent in high-grade carcinomas, but no correlation with survival was demonstrated [6].

The analysis performed in the present paper showed that the presence of positive locoregional lymph nodes has no impact on overall or disease-free survival. This feature may explain the previously reported good oncological results obtained with local resection of NET of the ampulla of Vater [29, 30]. The only factors that seem to be negatively correlated with patient prognosis upon univariate analysis are local invasiveness of the tumour (into the pancreas, retroperitoneum, serosa and other adjacent organs), large tumours (over 2 cm) and high-grade/G3 neuroendocrine carcinoma.

The present study has some limitations. First, the survival analysis is limited by the small number of patients, precluding a multivariate analysis. Furthermore, a relatively large number of patients had high-grade neuroendocrine carcinomas, a feature that could explain a worse long-term prognosis (compared to previous reports) [1, 7]. The retrospective design of this study may be associated with problems in data collection. Additionally, heterogeneity in the follow-up of the patients included in the survival analysis may be a confounding factor, mainly in regard to the long-term estimates of disease-free and overall survival.

## Conclusions

Although NET of the ampulla of Vater represents a rare pathology, it has been increasingly reported in the last years. The WHO classification seems to accurately predict prognosis, while the ENETS or UICC staging systems have limited value (especially in regard to lymph node metastases). However, NET limited to the ampulla of Vater (without local invasion) seems to have a better prognosis, as do tumours smaller than 2 cm.

Radical surgery (i.e. pancreaticoduodenectomy) should be the standard approach in most patients with NET of the ampulla of Vater, due to the high incidence and poor accuracy of preoperative and intraoperative assessments of lymph node metastases. The low accuracy of preoperative biopsies in the diagnosis of NET and the difficulty of differentiating NET from adenocarcinomas by frozen section analysis are also strong arguments for an aggressive surgical approach.

Although the overall and disease-free survival rates do not appear to be influenced by the presence of lymph node metastases, locoregional lymphadenectomy should be performed routinely because this procedure removes all the potential tumour-bearing tissue.

## Abbreviations

NET: Neuroendocrine tumours
WHO: World Health Organisation
TNM: Tumour–nodes–metastasis
CT: Computer tomography
MRI: Magnetic resonance imaging
ENETS: European Neuroendocrine Tumour Society
UICC: International Union Against Cancer
